# Molecular mechanisms of HPV mediated neoplastic progression

**DOI:** 10.1186/s13027-016-0107-4

**Published:** 2016-11-25

**Authors:** Rashmirani Senapati, Nihar Nalini Senapati, Bhagirathi Dwibedi

**Affiliations:** 1 Virology Division, Regional Medical Research centre (ICMR), Nalco square, Chandrasekharpur, Bhubaneswar, 751023 Odisha India; 2 Department of Anesthesia, Dr RML Hospital, New Delhi, India

**Keywords:** HPV integration, Carcinogenesis, Neoplastic progression, Cervical cancer

## Abstract

Human Papillomavirus is the major etiological agent in the development of cervical cancer but not a sufficient cause. Despite significant research, the underlying mechanisms of progression from a low-grade squamous intraepithelial lesion to high grade squamous intraepithelial lesion are yet to be understood. Deregulation of viral gene expression and host genomic instability play a central role in virus-mediated carcinogenesis. Key events such as viral integration and epigenetic modifications may lead to the deregulation of viral and host gene expression. This review has summarized the available literature to describe the possible mechanism and role of viral integration in mediating carcinogenesis. HPV integration begins with DNA damage or double strand break induced either by oxidative stress or HPV proteins and the subsequent steps are driven by the DNA damage responses. Inflammation and oxidative stress could be considered as cofactors in stimulating viral integration and deregulation of cellular and viral oncogenes during the progression of cervical carcinoma. All these events together with the host and viral genetic and epigenetic modifications in neoplastic progression have also been reviewed which may be relevant in identifying a new preventive therapeutic strategy. In the absence of therapeutic intervention for HPV-infected individuals, future research focus should be directed towards preventing and reversing of HPV integration. DNA damage response, knocking out integrated HPV sequences, siRNA approach, modulating the selection mechanism of cells harboring integrated genomes and epigenetic modifiers are the possible therapeutic targets.

## Background

Cervical cancer is the fourth most common cancer in women worldwide [1]. It is well known that high-risk HPV is the main etiological agent for this infectious viral carcinoma. Human papillomaviruses are small (50 nm) double-stranded DNA viruses composed of a genome of 8kilobase pair, enclosed inside a non-enveloped capsid protein. The genome includes three portions: (a) early genes (E1, E2, E4, E5, E6, E7) those regulate the vegetative and productive phase of viral life cycle;(b) late genes (L1, L2) which encode the capsid protein and (c) a non-coding regulatory region called long control region (LCR) involved in the regulation of viral replication and transcription [2].

Among 184 different HPV genotypes, only 40 diverse types can infect anogenital region which can be classified into 3 classes based on their oncogenic potential. HPV16, 18, 31, 33, 35, 39, 45, 51, 52, 56, 58, 59, 68, 73 and 82 are included in high-risk group while HPV6, 11, 40, 42, 43, 44, 54, 61, 70, 72 and 81 are included in low-risk group whereas HPV 26, 53 and 66 belong to the group of intermediate risk [3, 4]. Compelling evidence supports the high prevalence of HPV16 and 18 in the high-grade cervical lesion and considers these types to be the most potent carcinogenic viruses [5].

Based on the histopathological features cervical cancers are classified as squamous cell carcinoma (SCC), adenocarcinoma and adenosquamous carcinoma. Squamous cell carcinoma is the most common type of cervical cancer. The precancerous lesions which progress to SCC are called cervical intraepithelial neoplasia (CIN) or squamous intraepithelial lesion (SIL) which is classified according to the grade of the lesion [6]. A productive HR-HPV infection may develop into low-grade SILs (LSILs) which are nonmalignant bearing the low risk of progression to malignancy and corresponding to CIN1 [7]. The high-grade SILs (HSILs) comprise abortive virus infections in which there is deregulated expression of HPV early genes in basal epithelial cells, a greater risk of progression to invasive disease and corresponding to CIN2/3 [6]. Most of the HPV infections are subclinical and only a small fraction of HR-HPV infections produces early epithelial lesions [8, 9] and a more modest fraction of those lesions progress to higher grade lesion and invasive cancer. The mechanisms behind the progression of neoplastic lesions have not clearly understood. However, several viral and host factors and their interactions with each other have been proposed as potential candidates of carcinogenesis. In this review, we take a comprehensive look at the current understanding of molecular mechanisms behind the process of HPV-induced carcinogenesis with relevance to cervical cancer progression.

## Entry of HPV and life cycle in cervical epithelial cell

HPV, an epitheliotropic virus, infects basal epithelial cells of the squamous-columnar junction of the cervix. The virus makes its entry into the basal epithelial cells through micro-wounds or micro-abrasions. Hence early age first sexual debut, coarse sex and other sexually transmitted infections (STIs) promote virus entry and infection [10]. Heparan sulfate proteoglycans (HSPG) found in the extracellular matrix (ECM) on the cell surface are thought to be the initial receptors of HPV VLP [11, 12]. α6-integrin and laminin-5 play an important role as co-receptors for an efficient viral infection [13]. Initial attachment of HSPG moieties to L1 facilitates the conformational changes in L2 [14, 15]. Subsequently, the L2 protein is cleaved by furin on the cell surface at a consensus cleavage site that is conserved among all papillomaviruses [16]. Entry of virus into a host is very slow (up to 12 h), possibly due to conformational changes in capsid and receptors [17].

After a successful binding to the receptor, virus is internalized into the cell by clathrin or caveolae-mediated endocytosis [18, 19]. The viral genome enters into the nucleus through nuclear envelope breaks instead of nuclear pore. Then it localizes the ND10 bodies inside the nucleus [20–22]. Once entered the nucleus, HPV replicates to a low copy number (10–200 copy number/cell) during the initial amplification and establishment of infection [23]. As the proliferating cells (harboring HPV genomes) undergo transition through differentiation, the mode of viral genome replication switches to support productive viral genome amplification concomitant with increased levels of the E1 and E2 replication proteins [24]. In the terminally differentiated layer of epithelium L1 and L2 capsid proteins are expressed and viral particles are assembled. The virions are sloughed off with the dead squamous cells of the host epithelium for further transmission [25].

## Mechanism of HPV integration

Although integration is not a part of the normal HPV life cycle, high-risk HPV (HR-HPV) DNA is often integrated into the human genome in cervical SSC tissue sample [26–30]. It has been proposed that integration can be an early event associated with LSIL to HSIL progression [31–34], so expected to be a biomarker in cancer progression. However, the current understanding of the mechanism of integration is unclear.

Unlike retroviruses in which protein integrase facilitates their integration into the host genome, HPV encodes no such protein for this purpose. The site of integration is distributed throughout the genome as chromosomal fragile sites where DNA double strand breaks are failed to repair [35–37]. Integration hotspots in various genomic regions such as 3q28, 17q21, 13q22.1, 8q24.21, and 4q13.3 are reported by various researchers [36]. DNA damage is often induced by oxidative molecules such as reactive Oxygen species, reactive nitrogen species [38, 39] and HPV proteins E1, E6, &E7 [40–43].

Cell evolved with a special pathway to repair the continuous exogenous and endogenous damage in DNA named as DNA damage response (DDR). DDR plays a crucial role in preparing the cell to continue cell division. Once the damage is repaired, the cell cycle checkpoints are mitigated and cell continues to divide. If unrepaired cell undergoes apoptosis. However, unrepaired break points are the prerequisites for integration to occur.

Viral oncoproteins play a role in combating the downstream consequences of DNA damage response (DDR) in various ways. This favors in maintaining the breakpoints in host chromosomes required for integration. E6 and E7 oncoproteins disrupt cell cycle checkpoint control by inhibiting CDKs inhibitors [P21, P27] and degrading P53 [44–46]. HPV-16 E7 oncoprotein attenuates the DNA damage checkpoint response by accelerating the proteolytic turnover of claspin, a critical regulator of the ATR/CHK1 signaling axis and DNA damage checkpoint recovery in the G2 phase of the cell cycle [47]. HPV oncoproteins relax G1-S checkpoint control to induce unscheduled entry into S-phase and promote S-phase–like milieu conducive for viral genome replication in differentiated human keratinocytes [48, 49]. P53 is degraded by E6 oncoprotein and it is required for sensing base excision repair machinery and repairing of oxidative damage [50]. Thus, damage response fails to pose apoptotic threats to cell and allows replication to produce rearranged host genome with multiple breakpoints.

Interestingly, HPV takes advantage of this damage response pathway for its own replication and produces an ample number of episomal HPV which possibly increase the availability of more HPV DNA for integration into the host DNA. The DNA break-induced DDR triggers the accumulation of the factors for replication factories at the replication foci [43, 51]. Importantly, in the differentiated cells, this can occur in the G2 phase of the cell cycle where there is no competition from host DNA synthesis which is an additional advantage.

DNA damage response acts as a driving force throughout viral replication. Presence of ND10 at DNA damage region indicates the link between initial amplification of viral DNA with DDR [52]. It showed that during the initial amplification of viral DNA, the ATR-dependent DNA damage response engages at the HPV 18 replication centers [53]. Besides the initial amplification, DDR plays an important role to maintain the viral replication [54, 55]. The implication of DDR machinery in maintaining replication could be presumed by considering the association of CFS with ERD4 which helps to tether the viral genome with host DNA [56, 57].

The occasional association of Head-to-tail tandem repeats in the chromosome of cervical cancer cells [58, 59] leads to a plausible explanation that a linear concatemeric HPV genome is synthesized in cells by a rolling circle mechanism of replication and integrates into the host chromosome [60]. Upon induction of differentiation, genome switched to rolling circle mechanism of replication whereas in undifferentiated cells bidirectional replication occurs. The homologous recombination machinery is recruited to the regions of double strand breaks only and it is generated through collapsed replication fork during replication of viral genome [61] which might be induced by HPV E1. Double strand break in viral genome may be induced by HPV E1 during replication and homologous recombination [43, 51, 53, 61]. It is reported that DDR is involved in maintaining the double strand break in the viral genome [62, 63]. Failure to re-circularize the breaks produces linear HPV DNA with the double strand break which possibly enhances the scope for integration.

Proximity between host and HPV genome is required so that the fusion between virus and host could be completed. During the HPV life cycle, HPV genome tethers to host chromatin by HPVE2-BRD4 complex for partitioning of genomes to daughter cells [57]. E2-BRD4 complexes with acylated histones are observed at CFS region [56]. The association of BRD4 with the chromosomal fragile region (common site of integration) or the region of DNA damage leads to the presumption that BRD4 may play an important role in increasing the mechanistic feasibility of integration by promoting tethering of viral DNA with host genome at the region of DNA damage. However, there is no literature available to explain the role of BRD4 in HPV integration or any association of genome tethering with viral integration.

To complete the process of integration, fusion between host and HPV genome with DSB is expected to be accomplished through a recombination directed repair mechanism. Initially, nonhomologous end joining (NHEJ) is thought to be implicated in HPV integration [64]. According to a recent report, the presence of microhomologous sequences near the integration breakpoints indicate a homology-mediated DNA repair pathway during the fusion of human and viral DNA [65]. Although sequence homology is observed at the integration site, it is not perceived to be a prerequisite. There are two types of integration events [58]. In type-1 integration, a single copy of the viral genome gets integrated while in type-2, a concatamer of viral genome integrates into the host chromosome.

**Fig. 1:**
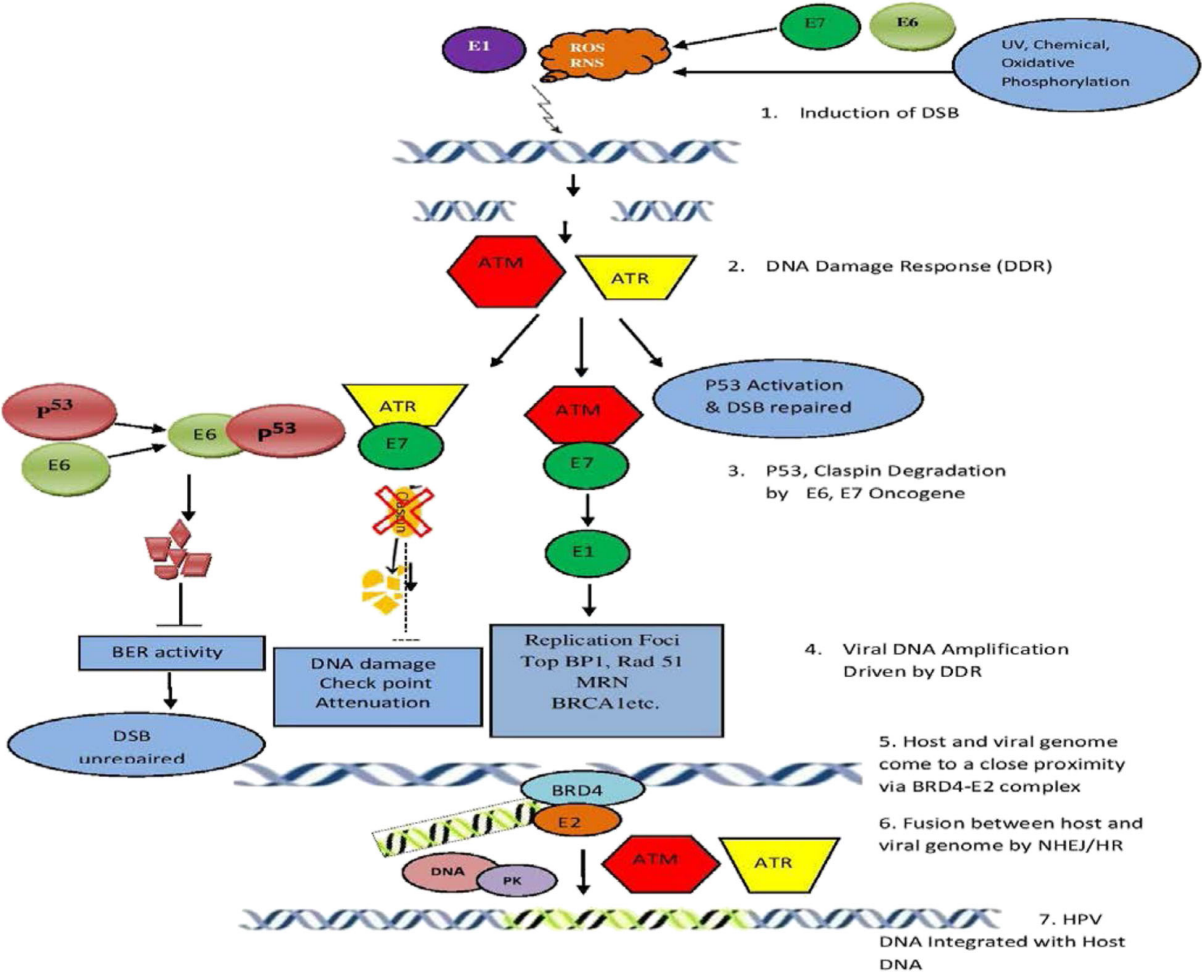
Conceptual model of viral integration. 1. Induction of DSB by ROS/NOS/Viral protein 2. DNA damage induces DDR (DNA damage response), ATM/ATR and P53 get activated to repair the damage. 3. HPV oncogenes deactivate the normal function of DDR molecules and DNA damage failed to be recognized. E7 degrades claspins and attenuate DNA damage checkpoints, while E6 degrades p53 and base excision repair gets suppressed so that the genomic DNA remains unrepaired and cell cycle proceeds. 4. Virus utilizes the DDR machinery for its replication which increases the availability of episomal DNA for integration. Breaks in the circular viral DNA may occur due to replication stress. 5. Virus and host genome come to a close proximity mediated by the BRD4-E2 complex. 6. Fusion between host and viral genome is accomplished either by Nonhomologous mediated end joining or homologous recombination repair pathway

Summarizing the above literature, the integration is started with DNA damage, induced either by oxidative stress or HPV protein and the subsequent steps are driven by the DNA damage responses (Fig. 1). Virus uses the DDR machinery to promote viral amplification while the viral oncoproteins render the cells to overcome the downstream consequences of damage response. Breaks in HPV DNA are introduced possibly during the replication of virus which might be induced by E1 and these breaks fail to get repaired. Availability of ample viral episomes to the host genome with multiple DSB enhances the possibility of integration. Proximity between viral and host genome by E2-BRD4-mediated tethering could possibly increase the mechanistic feasibility of viral integration. Finally, the fusion between both the genome via either homologous or nonhomologous recombination is regulated by the DNA damage response pathway (ATM/ATR and DNA-PK pathways) [66].

## Role of HPV integration in carcinogenesis

Integration of HPV influences both viral and host genome. Viral genes such as E1, E2, E5, and L2 region get disrupted by integration and consequently E6 and E7 oncogenes overexpressed in the absence of E2 repressor protein [67]. Overexpression of E6/E7 results in cell cycle deregulation and promotes several other pathways leading to carcinogenesis. Integrated HPV DNA gives a selective growth advantage to the cell as compared to the episomal counterparts and it is attributed to the presence of host polyadenylation tail and loss of apoptotic protein E2 [68]. Episomal loss due to IFN response is considered to be a determinant for the selection of integrant is being supported by many studies [69–71].

Genomic instability is the hallmark of cancer. A recent study reported a direct association between HPV integration and host genomic instability. It showed HPV integration driven chromosomal rearrangements which include deletions, translocations and inversion in the genomic regions flanking HPV integrants. The study suggested that integration is a possible insertional mutagenesis event which could lead to a specific change in gene expression at the site of integration [72]. This hypothesis is strengthened by another study showing a high level of host gene expression at HPV integration sites [73] as compared to the expression of the same genes in tumors without viral integration at the same sites [73]. Genes nearer to the integration sites with high expression level are reported to be MYC, ERBB2, GLI2, TNIK, NR4A2, PROX1, EIF2C2, FAM179B, and SERPINB4, RPS6KB1, MAFA, PARN, EGFL7, SNIP1, POC1B, and BCL11B [73]. Integration impacts the host genome by amplification of oncogenes and disruption of tumor suppressor genes as well as driving inter- and intra-chromosomal rearrangements.

## Inflammation and oxidation play role as cofactors in neoplastic progression

Inflammation contributes to carcinogenesis via promoting oxidative damage, cell proliferation, invasion, and metastasis, inhibiting apoptosis and secreting immune suppressors [74, 75]. Chronic inflammation may lead to DNA damage by enhancing cellular ROS, NOS and foster the progress of low-grade to the high-grade lesion.

The interplay of viral oncoproteins and inflammatory cytokines may lead to develop persistent infection by continuous immune evasion which promotes progression of the lesion and ultimately leads to malignancy. Being double standard DNA, HPV genome is recognized by TLR-9 present in the DCs, macrophages, and NK cells. Interferon responses possibly facilitate selection of integrants and overexpression of E6/E7 through enhancing the depletion of episomal DNA [76, 77]. Once the early genes E6/E7 are expressed, TLR9 downregulated and IFN response impaired resulting in a conducive milieu for immune evasion and persistent infection [78–81]. Impairment of interferon pathway leads to down-regulation of MHC I expression on a cell surface. As a result, CD8-cytotoxic T cell can’t get activated and unable to initiate T cell response. This process of continuous immune evasion may ultimately lead to the persistence infection and carcinogenesis. Overexpression of E6 and E7 in cell imparts resistance to TNF- α induced apoptosis and antiproliferative effect [82–84]. NF-κB, a key modulator for chronic inflammation, is activated by PDZ binding domain of HPV E6. Diminished cell proliferation and rapid apoptosis observed in cells infected with mutant PDZ binding domain [83] which suggests that NF-κB involve in E6/E7 mediated carcinogenesis.

An increased Oxidative stress in cell attributes to inflammation, chemical stress, UV exposure, oxidative phosphorylation etc. Oxidative stress leads to DNA damage and viral integration which have been described earlier. Besides, DNA damage, proteins are also prone to oxidative modifications such as carbonyls and nitrotyrosine adducts, as observed in conditions like aging and cancer [85, 86]. Accumulation of oxidative proteins may lead to the deregulation of important pathways leading to carcinogenesis. Increased levels of carbonyl in cytokeratin-6, actins, cornulin, retinal dehydrogenase and GAPDH have been observed in HPV16 dysplasia and neoplastic tissue [87]. However, the effect of protein oxidation on cellular transformation and carcinogenesis is not yet been clearly understood. This phenomenon has a greater implication in improving clinical protocol for screening and prognostic evaluation of cervical cancer. The role of HPV proteins to generate oxidative stress is yet to be explored. However, there is limited data demonstrating the role of E6 and E2. No experimental data is available on the impact of OS on the regulation and function of E4, E5, L1 and L2. The impact of viral mediated oxidative stress on viral life cycle and carcinogenesis needs to be explored.

Inflammation and oxidative stress could be the cofactors in stimulating viral integration and deregulation of cellular and viral oncogenes during the progress of cervical carcinoma.

## Epigenetic modification in cervical cancer

Genetic and epigenetic modifications at regulatory region of HPV may contribute to the overexpression of viral oncogene at episomal state [88–91]. Association of cervical carcinoma progression with the methylation of L1, L2, E5, E2 and L2 region of different HR-HPV genotypes is supported by many epidemiological and in vitro studies [92–94]. It is important to consider the methylation in the long control region (LCR) as it regulates the transcription and replication of HPV genes. The LCR is the region of the E2 binding site which possesses CpG sites for potential methylation, resulting in inhibition of E2 function. It was initially reported that HPV16 LCR methylation in the promoter and enhancer region decreases with severity of lesion [95]. Methylation is found to be more common in invasive cervical carcinoma and cervical intraepithelial neoplasia (CIN) III than in CIN I-II (84.6% and 46.2% vs. 29.4%, respectively) as reported by Hong et al. in 2008(97). In a later study, it is revealed that CpG hypermethylation of the HPV16 LCR increase with the severity of the cervical neoplasia [low grade squamous intraepithelial lesion (LSIL): 5.9%; high-grade squamous intraepithelial lesion (HSIL): 33.3%; squamous cell carcinoma (SCC): 53.3%] [96]. LCR methylation is also reported in 71.4% of asymptomatic infection cases. However, a clear association of LCR methylation with neoplastic progression has not yet been established.

The important histone modifications such as methylation and acetylation at H3 lysine 27, H3 lysine 9 and H4 lysine 20 positions significantly contribute to the regulation of viral gene expression [97, 98]. In the context of the neoplastic progression, acetylation of H3 increases as the cell progresses phenotypically from normal to SCC in both episomal and integration mediated carcinogenesis. H3 lysine 27 trimethylation and H3 lysine 9 trimethylation markers decrease with the neoplastic progression in integration mediated carcinogenesis [99, 100].

Like other cancers, epigenetic modification is a prominent feature of cervical cancer. A wide range of host genes involved in cell cycle regulation, apoptosis, DNA repair and WNT pathway often undergo epigenetic modification in cervical cancer. DcR1 and DcR2 are the apoptotic genes reported being hypermethylated in invasive cancer [101] suggesting that cervical cells get a growth advantage by downregulating decoy receptor expression [102]. Other examples include the downregulation of p73 in cervical cancer due to promoter hypermethylation [103] and correlation of hypomethylated hTERT with disease prognosis [104]. RASSF1 is a key gene involved in the apoptotic signaling pathway which is downregulated in cervical cancer via methylation is being reported in many studies [105, 106]. Downregulation of CADM1 gene leads to metastasis and cancer progression. Promoter hypermethylation is associated with decreased expression of CADM1 in high¬grade CIN and SCC [107]. Frequency and density of CADM1 methylation increase with severity of dysplasia [107]. Hence CADM1 is suggested to be a possible epigenetic diagnostic marker in predicting the risk of cancer progression. WIF1, APC, and CDH1 are significantly hypermethylated in cervical cancers [108] which are the key regulators of Wnt/β-catenin pathway. Increased methylation in DAPK1, RARB, TIMP3, CCNA, and FHIT is associated with cervical cancer and low or no methylation is observed in LSHL lesion and normal cytology [109–111]. Host genes are reported as methylation marker to distinguish abnormal lesion from the normal one. CCNA1 could be a methylation marker to distinguish normal lesion from high-grade lesion while CCNA1, hTERT1, hTERT2 and TWIST1 could distinguish cervical cancer from normal and precancerous stage [112].

Apart from demographic and lifestyle factors [113], methylation of HPV and host gene are likely to be influenced by HPV integration and multiple genotypes. Methylation is higher in single type as compared to infection with multiple HPV genotypes [113]. Moreover, host methylation pattern is reported to be integration dependent [114].

## Association of miRNAs in carcinogenesis

miRNAs are the noncoding RNAs that regulate expression of the target mRNAs at the post-transcriptional level. miRNAs play a significant role in initiating and promoting the process of carcinogenesis by inducing cellular proliferation, apoptosis and genomic instability [115].

Carcinogenesis process is influenced by both up regulation and down regulation of miRNAs. Increased expression of certain mi-RNAs (*viz*., miR-886-5p, miR-10a, miR-141, miR-21, miR-135b, miR-148a, miR-214 and miR-106b) plays vital role in cervical cancer progression as they are involved in regulation of cell proliferation, apoptotic pathway or cell adhesion [116–122]. Down regulation of let-7c, miR-124, miR-126, miR-143, and miR-145 regulates the expression of oncogenes.

The role of miRNAs via E6 and E7 oncogenes in regulating cell cycle progression, senescence, and apoptosis have been reported by several studies [123]. In cervical cancer, p53 is the most important target of E6 oncogene. Hence, expression of all the miRNAs regulated by P53 is thought to be regulated by E6 oncogene function. For example, miR-23b, miR-34a, and miR-218 are down-regulated by E6 oncogene via degradation of p53 [124, 125].

E7 induced overexpression of miR-15/16 via E2F1 deactivation resulting down-regulation of c-myc or c-myb. E7 enhances the expression of miR-15a/miR-16-1 which inhibits cell proliferation, survival, and invasion. miR-203 is downregulated by E7 via MAPK/PKC pathway [125].

Considering the significant changes in miRNA expression during the progress of normal cervical epithelium to high-grade CIN lesions and ultimately to SCC or AdCAs [126], the microRNA expression profiling may help in preparing a specific genomic and/or transcriptomic signature of cervical tissue. It could be used as possible diagnostic and prognostic biomarkers for cervical carcinoma. However, more study is required for its proper validation in clinical samples.

## Association of HPV variants in cervix cancer pathogenesis

Sequence variations in HPV genome would lead to the evolution of variants with differences in infectivity and pathogenicity. The attribution of HPV variants to cancer development could be due to the difference in the capacity to cause persistence infection and regulation of viral oncogene expression. The association of HPV16, HPV31, HPV52 and HPV 58 variants with high oncogenicity, persistence, and progression of infection is reported in many studies [127–130].

Several epidemiological data from the regions with high prevalence of cervical carcinomas such as Latin America, Africa, and Asia have a high prevalence of sublineages AA and Af [131]. Non-European HPV16 variants such as Af1 and AA were found at an increased frequency in invasive lesions while it is low in high-grade lesion [131]. A Recent study in South Mexican population suggests that oncogenic HPV 16 AA-a strain carries a higher risk to progress cervical cancer. The association of sub-lineage AA with persistent infection and the risk of cancer development are higher than that of EUR sublineage [130, 132, 133]. These data collectively shows a strong association of HPV 16 AA variants with persistent infection and development of cancer while other non-European variants have a lesser degree of association. Hence HPV16AA variant is considered to be a potential carcinogenic strain. Only three amino acid changes within the E6 of HPV16 AA are connected to this augmented carcinogenic ability [129]. Enhanced ability of AA E6 variants in promoting cellular immortalization, migration, invasiveness and ability to undergo transformation to resilient phenotypes have been demonstrated using in vitro model of retrovirally transduced primary human foreskin keratinocytes mimicking persistently infected and post-integrated keratinocyte system [134, 135]. Tumorigenic potential of full-length HPV 16 AA E6 variant has also been demonstrated in organotypic tissue culture model [136].

Besides HPV16, the variants of other genotypes (HPV 18, HPV 31, HPV 52 and HPV 58) have also been reported to be associated with the risk of persistent infection and development of cervical cancer. Xi et al. demonstrated that HPV 18 E/AsAi variants had a 2-fold risk of developing CIN3 as compared to the African variants [137]. Studies also suggest that the HPV 18 AsAi lineage (A1/A2) is 4-fold more common in Adenocarcinoma than the E lineage [138].

Literature shows conflicting data regarding the association of HPV 31 variants with the risk of persistence infection and progression. It is reported that HPV 31 lineage C is more persistent than that of A and B lineage [139, 140]. However, other literature reflects that A and B lineage are mostly associated with persistent infection and progression of diseases. More particularly lineage B was associated with CIN 3 as compared to C or B [139, 141].

In Case of HPV 52 variants, it is reported that B lineage is associated with a 7.6 fold risk [95% CI: 1.3–44] as compared to lineage C, in women with HPV52 as a single infection [142]. However, A1 lineage is more common in CIN 2/3. For HPV 58, A lineage is associated with CIN3+ as compared to B, C and D. Geographical distribution of HPV variants, epidemiological risk factors and complex interaction of viral variants with host genetics need to be considered while designing epidemiological studies with HPV variants and cancer pathogenesis.

## Conclusion

**Fig. 2:**
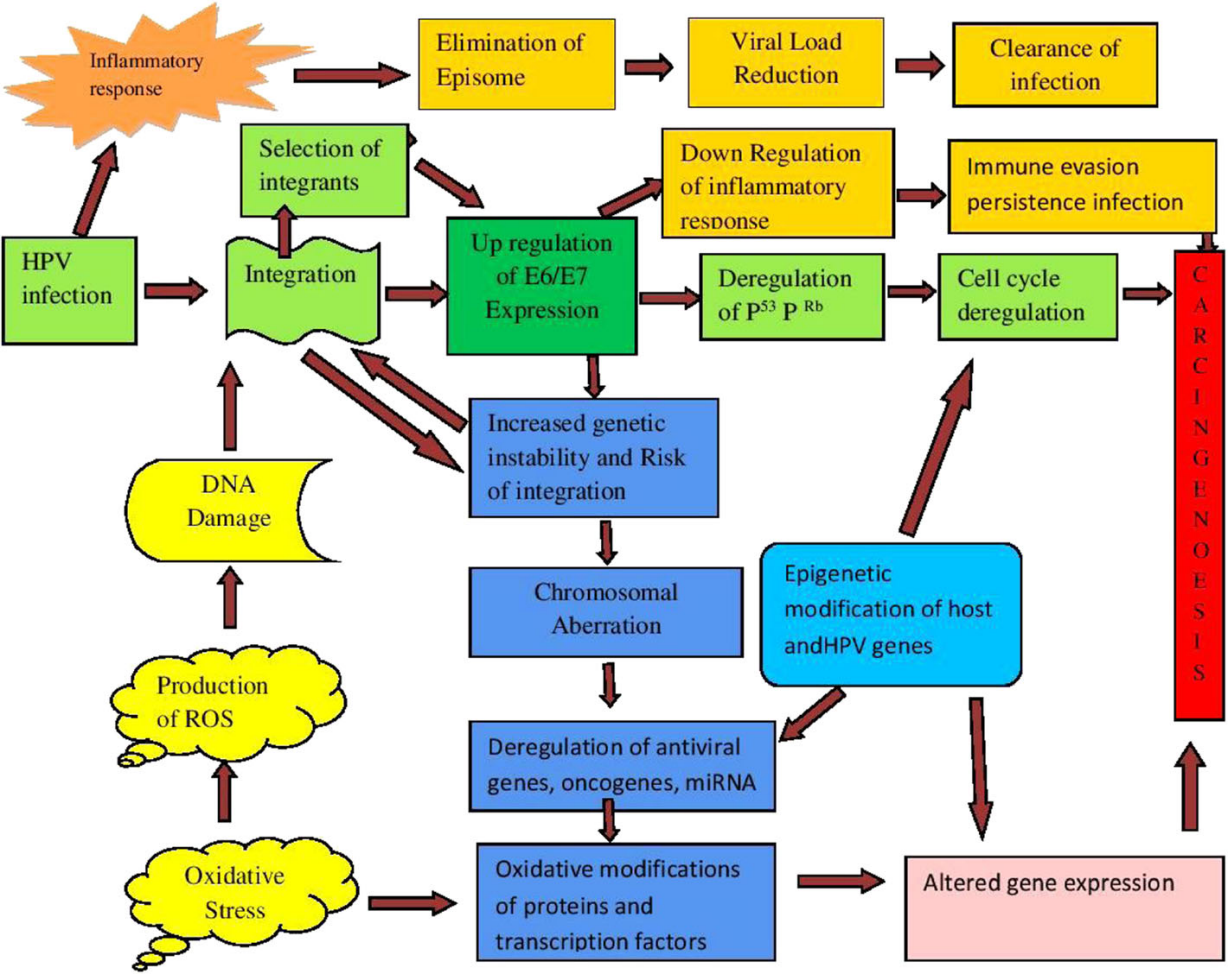
HPV-driven carcinogenesis: a multistep molecular mechanism of host-viral interaction. The initial outcome of carcinogenesis is modulated by both viral (high-risk versus low-risk HPV types, HPV integration) and host factors (inflammatory response, oxidative stress). Inflammatory response upon initial infection such as IFN response plays role in reducing episomal HPV resulting clearance of infection. Integration of HPV is initiated with DNA damage. The IFN induced loss of episomal HPV and down-regulation of E2 leads to the selection of cells with integrated HPV genomes expressing higher levels of E6 and E7. Once the early genes E6 &E7 are expressed, TLR9 downregulated and IFN response impaired, resulting a conducive milieu for immune evasion and persistent infection. Upregulation of E6/E7 increases genetic instability and chromosomal rearrangements that increase the risk of integration. Overexpression of E6/E7 leads to deregulation of the cell cycle via p53 & Rb degradation, deregulation of oncogenes and miRNAs expression. Epigenetic and genetic modification in viral and host genome leads to the deregulation of E6 &E7 oncogenes, and host tumor suppressor genes that lead to carcinogenesis. Oxidative modification of TFs also leads to altered gene expression and carcinogenesis

This review has described the important molecular mechanisms of HPV mediated carcinogenesis in human cells. After a successful persistent infection of HR-HPV variants, neoplastic progression is accomplished through several routes (Fig. 2). Events of fundamental importance leading to neoplastic changes during the progression of cervical carcinoma are persistent infection, overexpression of viral oncogene and host genomic instability. These events could be possibly driven by host immune responses, viral integration, and host/viral epigenetic modifications.

Current HPV vaccination strategy targets at preventing the infection of few restricted HPV genotypes in uninfected individuals. There is also a lack of treatment strategy for clearance of viral infection. In this scenario future research focusing prevention and reversal of HPV integration and epigenetic modifications may provide a way to arrest neoplastic progression. A clear understanding of the role of DNA damage response in viral integration may consider DDR as a target to prevent HPV integration. Knocking out integrated HPV DNA sequences by gene editing and SiRNA approach to reducing viral oncogene transcripts and modulating the mechanisms of selection of cells harboring integrated HPV genome may be considered in this regard. The implication of epigenetic modifiers as a therapeutic approach could be a major future strategy.

## Abbreviations

AP-1: Activator protein 1
ATM: Ataxia telangiectasia mutated
ATR: Ataxia telangiectasia and Rad3-related protein
Bax: BCL2-associated X protein
BRCA1: Breast cancer type1
BRD4: Bromodomain-containing protein4
CADM1: Cell adhesion molecule 1
CCNA1: Cyclin-A1
CDKs: Cyclin-dependent kinases
CEBPB: CCAAT/enhancer-binding protein beta i
CHK1: Checkpoint kinase 1
CIN: Cervical intraepithelial neoplasia
DAPK1: Death-associated protein kinase 1
DC: Dendritic cells
DcR1: Death receptor 1
DDR: DNA damage response
DNA-PK: DNA-dependent protein kinase
DNMT1: DNA (cytosine-5)-methyltransferase 1
DSB: Double strand break
FHIT: Fragile histidine triad protein
GADPH: Glyceraldehyde 3-phosphate dehydrogenase
GRE: Glucocorticoid response element
HDAC: Histone deacetylases
HPV E/As/Ai variant: HPV European/Asian/American Indian variant
HPV: Human papillomavirus
HSIL: High-grade squamous epithelial lesion
HSPG: Heparan sulfate proteoglycans
hTERT: Human telomerase reverse transcriptase
IFN: Interferon
LCR: Long control region
LSIL: Lowgrade squamous epithelial lesion
MCP-1: Monocyte chemoattractants protein
MRN: Mre II, Rad50, NbsI
ND10: Nuclear bodies 10
NF-1: Neurofibromatosis type-1
NF-κB: Nuclear factor kappa-light-chain-enhancer of activated B cells
NGS: Next generation sequencing
NHEJ: Nonhomologous end joining
NK: Natural killer cells
Oct-1: Octamer transcription factor-1
OS: Oxidative stress
P53: 53 Kilodalton phosphoprotein
PRB: Retinoblastoma protein
RARB: Retinoic acid receptor, beta
RASSF1: Ras association domain-containing protein 1
SiRNA: Small interfering RNA
TFs: Transcription factors
TIMP3: Metalloproteinase inhibitor 3
TLR: Toll-like receptor
TOPBP1: DNA Topoisomerase-2 binding protein 1
TWIST1: Twist-related protein 1
URR: Upstream regulatory region
VLP: Virus-like particle
WIF1: Wnt inhibitory factor 1
WNT: Wingless-related integration site
YY-1: Yin Yang 1
